# Paraaortic lymph node metastasis in endometrial cancer patients: a comprehensive analysis of rates, survival outcomes, and risk factors through systematic review and meta-analysis

**DOI:** 10.3389/fonc.2024.1490347

**Published:** 2024-10-31

**Authors:** Ling Han, Yali Chen, Ai Zheng, Xin Tan, Hengxi Chen

**Affiliations:** ^1^ Department of Obstetrics and Gynecology, West China Second University Hospital, Sichuan University, Chengdu, China; ^2^ Key Laboratory of Birth Defects and Related Diseases of Women and Children (Sichuan University), Ministry of Education, Chengdu, China; ^3^ Day Surgery Department, West China Second University Hospital, Sichuan University, Chengdu, China

**Keywords:** endometrial cancer, paraaortic lymph node metastasis, risk factor, survival, meta-analysis

## Abstract

**Introduction:**

This study aims to explore the incidence of different metastatic patterns in paraaortic lymph nodes and their corresponding survival outcomes in patients with endometrial cancer, as well as to identify the associated risk factors of such metastasis.

**Material and methods:**

PubMed, Embase, Cochrane Central Register of Controlled Trials, International Clinical Trials Registry Platform, and Clinical Trials.gov were searched from inception to February 10, 2024.The analysis was conducted using R version 4.2.3.

**Results:**

A total of 47 studies involving 33,425 endometrial cancer patients were analyzed. Meta-analysis results revealed that the rate of isolated paraaortic lymph node metastasis, where pelvic lymph nodes were negative but paraaortic lymph nodes were positive (PLN-PAN+), was found to be 2.58% (95% CI 0.0195-0.0329). The rates for PLN+PAN- and PLN+PAN+ were notably higher at 8.54% (95% CI 0.0642-0.1092) and 8.37% (95% CI 0.0613-0.1090), respectively. For clinical stage I EC, the occurrence rate was 5.92% for PLN+PAN- (95% CI 0.0258-0.1032), 1.00% for PLN-PAN+ (95% CI 0.0081-0.0120), and 2.99% for PLN+PAN+ (95% CI 0.0188-0.0431). The survival outcomes indicate a decreasing trend from the PLN-PAN+ and PLN+PAN- groups to the PLN+PAN+ group. Additionally, the survival outcomes of patients with isolated paraaortic lymph node metastasis appear to be comparable to, or not inferior to, those of the PLN+PAN- group. The analysis indicated that pelvic lymph node metastasis (OR 16.72, 95% CI 10.03-27.86), myometrial invasion ≥50% (OR 5.18, 95% CI 3.09-8.69), lymph-vascular space invasion (LVSI) (OR 3.46, 95% CI 2.49-4.81), cervical invasion (OR 4.00, 95% CI 2.09-7.66), and non-endometrioid cancer (OR 2.39, 95% CI 1.17-4.86) were risk factors for paraaortic lymph node metastasis.

**Conclusions:**

Isolated paraaortic lymph node metastasis, though relatively rare, can still occur even in clinical stage I endometrial cancer. The survival outcomes of patients with isolated paraaortic lymph node metastasis appear to be comparable to, or not inferior to, those of the PLN+PAN- group. Even in patients with negative pelvic lymph nodes, careful consideration should be given to the possibility of paraaortic lymph node metastasis, especially in those with high-risk factors.

**Systematic review registration:**

https://www.crd.york.ac.uk/prospero/, identifier CRD42024503959.

## 1 Introduction

Endometrial cancer ranks as the fourth most frequently diagnosed cancer among women globally, with a mortality rate that has witnessed a 1% annual increase (1). In the United States, it stands as the most prevalent female genital tract cancer, accounting for 65,950 new cases and 12,550 deaths in the year 2022 alone (2). A significant proportion of endometrial cancer cases are detected at an early stage, confined to the uterus. The standard treatment for such cases involves surgical intervention, typically comprising hysterectomy, bilateral salpingo-oophorectomy, and may include lymphadenectomy (2).

Lymphatic metastasis represents the primary route for endometrial cancer spread and is a crucial prognostic factor affecting survival rates. Current surgical approaches for lymph node staging in endometrial cancer encompass a range of methods, including no nodal assessment, lymph node biopsy, sentinel lymph node mapping, and pelvic and paraaortic lymphadenectomy extending up to the renal vessels (3). Since the FIGO2009 update, Stage IIIC has been further classified into IIIC1 and IIIC2 based on the presence of positive pelvic and paraaortic lymph nodes (4). Stage IIIC2 specifically involves the coexistence of positive paraaortic pelvic lymph nodes with pelvic lymph nodes, or the presence of isolated paraaortic lymph nodes without involvement of pelvic lymph nodes. However, literature on the performance of paraaortic lymphadenectomy in early-stage endometrial cancer has yielded conflicting results, with some authors reporting no survival benefit (5, 6), while others argue that completing lymphadenectomy improves survival rates (7). The identification of molecular classification assists in determining the prognostic groups of patients, facilitates prompt decision-making, and enables the development of tailored strategies for each patient (8).

Accurate identification of lymph node status plays a crucial role in stratifying patient prognosis and formulating an effective treatment plan for endometrial cancer. However, identifying lymph node status in endometrial cancer, especially at early stages, poses challenges. While paraaortic lymphadenectomy aids in determining paraaortic status, its implementation is associated with prolonged surgery time and higher intraoperative complications. Moreover, while the adoption of sentinel lymph node sampling is recommended for its high detection and sensitivity rates and low false-negative rate in endometrial cancer (9), there is still concern regarding the regional coverage of paraaortic nodes, particularly with dye injection through the cervix, which could potentially lead to the oversight of paraaortic metastases (10).

The aim of this study is to investigate the incidence of various metastatic paraaortic lymph nodes pattens and their associated survival outcomes, as well as to identify the associated risk factors of such metastasis. This study holds great significance in assessing the impact of inadvertently omitting paraaortic lymph node dissection on survival and in exploring the risk factors associated with paraaortic lymph node metastasis.

## 2 Methods

### 2.1 Protocol registration

This meta-analysis was performed in accordance with the Preferred Reporting Items for Systematic Reviews and Meta-analyses (PRISMA) guidelines and was registered with the International Prospective Register of Systematic Reviews (PROSPERO) (CRD 42024503959) (11).

### 2.2 Eligibility criteria

All potentially eligible studies, including case-control and cohort studies published in English, were taken into consideration. The inclusion criteria comprised studies that met the following conditions: (1) Investigated metastatic lymph node patterns in endometrial cancer patients, encompassing pelvic lymph node metastasis without paraaortic lymph node metastasis (PLN+PAN-), paraaortic lymph node metastasis without pelvic lymph node metastasis (PLN-PAN+), and cases where both pelvic lymph nodes and paraaortic lymph nodes were metastasized (PLN+PAN+). (2) Examined predicting factors and/or survival outcomes of patients with metastatic paraaortic lymph nodes. Exclusion criteria: (1) Redundant publications. If data subsets were published in more than one article, only the largest sample size was included; (2) Incomplete data; (3) Conference abstracts and reviews.

### 2.3 Search strategy and study selection

PubMed, Embase, Cochrane Central Register of Controlled Trials (CENTRAL), International Clinical Trials Registry Platform (ICTRP), and Clinical Trials.gov were searched from inception to February 10, 2024. The reference lists of the published reviews and retrieved articles were checked for additional trials. Search terms were as follows: “endometrial cancer”, “endometrial carcinoma”, “para-aortic”, “paraaortic”, “nodal involvement”, “nodal metastasis”, “node metastasis”, “nodes metastasis”, “lymphatic metastasis”, “node involvement”, “nodes involvement”, “node positive”, “nodes positive”, “LN involvement”, “LN metastasis”, “LN positive”, “positive LV”, “positive lymph node”, “lymph dissection”, “lymphadenectomy”, “survival”, “outcome”, “prognosis”, “prognostic”, “recurrence”, “relapse”, “rate”, “isolated”, “factors”, “factor”.

Two researchers (HC and LH) independently screened the titles and abstracts to assess the eligibility of the studies. After initial selection, the full texts of all potential articles were independently read by two researchers (HC and LH) for further evaluation. Disagreements between authors were resolved by discussion with the XT.

### 2.4 Data extraction

Data extraction was carried out independently by two reviewers (LH and YC) in duplicate. The predefined extraction form encompassed the following variables: (1) first author and publication date; (2) study country; (3) study design; (4) quality assessment; (5) tumor pathologic type and stage; (6) number of patients with different metastatic lymph node patterns; (7) risk factors; (8) survival outcomes. Review authors were kept blind to institutions, sources of funding, and acknowledgments. A double data entry process was implemented.

### 2.5 Risk of bias assessment

Two reviewers (LH and YC) independently evaluated the quality of the included studies. Discrepancies were resolved through discussion, and in cases where consensus was not achieved, a third review author (AZ) was consulted. Cohort studies were assessed using the Newcastle–Ottawa Scale (NOS) based on three categories: selected cases, comparability of groups, and assessment of outcomes. Studies that received six or more stars were categorized as having high quality.

### 2.6 Statistical analysis

R version 4.2.3 (R Foundation for Statistical Computing, Vienna, Austria) was used for meta-analysis. The rates of positive lymph nodes were calculated with the Freeman-Tukey double arcsine transformation. Hazard ratios (HRs) and odd ratios (ORs) with 95% confidence intervals (CIs) were used to combine data concerning survival outcomes and risk factors. For studies reporting survival data only in the form of Kaplan-Meier curves, Engauge Digitizer 4.1 was used to extract survival data, and HRs and CIs were calculated according to the methods reported in literature. A p-value of <0.05 was considered statistically significant for the meta-analysis. Heterogeneity between studies was assessed using I² test: I²<30% denoted low heterogeneity, 30%<I²<50% denoted moderate heterogeneity, and I²≥50% denoted high heterogeneity. In cases of substantial heterogeneity, a random-effects model was employed to combine data; otherwise, a fixed-effects model was used. Publication bias was evaluated through the funnel plot, and statistical assessment was conducted using the Egger test. Results that could not be meta-analyzed were presented in a narrative format.

## 3 Results

### 3.1 Study selection and characteristics

**Figure 1:**
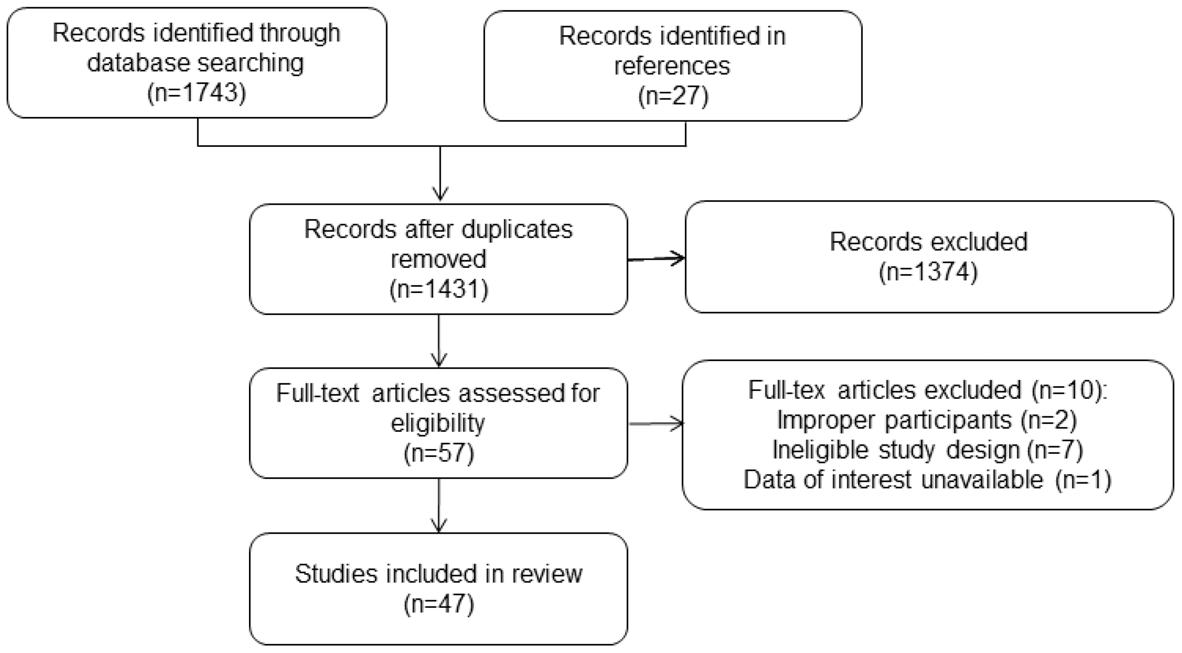
Study Selection Flowchart.

The study selection process was briefly depicted in **Figure 1**. After removing duplicates, 1273 articles were retrieved and screened based on their titles and abstracts. Later, 57 full texts were procured for further assessment, of which 10 articles were excluded after reading the full texts. Ultimately, the analysis included 47 studies with 33425 participants (12–58). The general characteristics of these studies were presented in **Table 1**. All included studies were retrospective studies and received a rating of six or more stars according to the NOS criteria.

**Table 1:** The basic characteristics of the studies included.

study	method of lymph node staging	total number	PLN+PAN-	PLN-PAN+	PLN+PAN+	tumor type	clinical stage
Abu-Rustum2009 (12)	Pelvic and paraaortic nodal dissection	640	52	7	61	All type	I-IV
Altay2015 (13)	Pelvic and paraaortic nodal dissection	173	12	7	19	All type	I-IV
Baiocch2017 (14)	Pelvic and paraaortic nodal dissection	255	15	7	21	All type	I-IV
Chang2011 (15)	Pelvic and paraaortic nodal dissection	203	10	14	5	All type	I-IV
Chiang2011 (16)	Pelvic and paraaortic nodal dissection	171	12	2	3	All type	I-IV
Guo2020 (17)	Pelvic and paraaortic nodal dissection	2767	1127	379	1261	All type	III-IV
Lee2009 (18)	Pelvic and paraaortic nodal dissection	22	1	2	0	Endometrioid type	I
Marchocki2023 (19)	Sentinel lymph node biopsy	612	49	5	14	All type	I-IV
Multinu2019 (20)	Pelvic and paraaortic nodal dissection	394	–	10	–	All type	I-III
Nasioudis2019 (21)	Pelvic and paraaortic nodal dissection	14398	–	230	–	All type	I
Nayyar2018 (22)	Pelvic and paraaortic nodal dissection	44	1	1	1	Endometrioid type	I
Odagiri2014 (23)	Pelvic and paraaortic nodal dissection	266	16	7	19	All type	III-IV
Ozsoy2003 (24)	Pelvic and paraaortic nodal dissection	58	2	1	2	Endometrioid type	I
Rathod2014 (25)	Pelvic and paraaortic nodal dissection	52	–	1	17	All type	I-III
Sautua2015 (26)	Pelvic and paraaortic nodal dissection	90	3	6	4	All type	NA
Somashekhar2021 (27)	Pelvic and paraaortic nodal dissection	210	17	15	29	All type	I-II
Suchetha2021 (28)	Pelvic and paraaortic nodal dissection	129	21	5	4	All type	I
Todo2017 (29)	Pelvic and paraaortic nodal dissection	380	25	9	30	All type	I-IV
Sueoka2015 (30)	Pelvic and paraaortic nodal dissection	502	27	15	38	All type	I-III
Togami2018 (31)	Sentinel lymph node biopsy	38	–	0	–	All type	I-IV
Tomisato2014 (32)	Pelvic and paraaortic nodal dissection	260	83	9	48	All type	I-IV
Türkmen2018 (33)	Pelvic and paraaortic nodal dissection	961	–	25	–	–	–
Vaizoglu2013 (34)	Pelvic and paraaortic nodal dissection	261	14	4	8	All type	I-IV
Li2021 (35)	Pelvic and paraaortic nodal dissection	4001	167	114	150	All type	I-IV
Widschwendter2018 (36)	Pelvic and paraaortic nodal dissection	111	12	3	15	All type	I-III
Yoon2010 (37)	Pelvic and paraaortic nodal dissection	131	–	2	4	Endometrioid type	I-III
Zhang2023 (38)	Sentinel lymph node biopsy or Pelvic and paraaortic nodal dissection	212	8	5	4	All type	I-III
William1987 (39)	Pelvic and paraaortic nodal dissection	621	36	12	22	All type	I
Dogan2011 (40)	Pelvic and paraaortic nodal dissection	165	12	2	5	All type	I-IV
Karube2010 (41)	Pelvic and paraaortic nodal dissection	355	–	7	–	Endometrioid type	I-III
Yoshikawa1997 (42)	Pelvic and paraaortic nodal dissection	173	10	2	18	All type	I-III
Kumar2014 (43)	Pelvic and paraaortic nodal dissection	425	39	11	37	All type	NA
Numanoglu2014 (44)	Pelvic and paraaortic nodal dissection	157	8	4	15	Endometrioid type	I-IV
Chen1983 (45)	Pelvic and paraaortic nodal dissection	74	3	3	5	All type	I
Morrow 1991 (46)	Pelvic and paraaortic nodal dissection	895	–	18	–	All type	I-II
Fujimoto2009 (47)	Pelvic and paraaortic nodal dissection	335	20	7	22	Endometrioid type	I-III
Larson1993 (48)	Pelvic and paraaortic nodal dissection	50	–	0	8	Endometrioid type	I-IV
Mariani2004 (49)	Pelvic and paraaortic nodal dissection	566	76	2	40	All type	I-IV
McMeekin2001 (50)	Pelvic and paraaortic nodal dissection	47	20	8	19	All type	III
Yokoyama 2009 (51)	Pelvic and paraaortic nodal dissection	63	6	4	8	All type	I-IV
Solmaz2015 (52)	Pelvic and paraaortic nodal dissection	516	37	4	26	Endometrioid type	I-III
Sari2017 (53)	Pelvic and paraaortic nodal dissection	641	28	15	47	All type	I-IV
Fotopoulou2015 (54)	Pelvic and paraaortic nodal dissection	128	8	4	15	All type	I-IV
Turan2011 (55)	Pelvic and paraaortic nodal dissection	204	19	5	21	All type	I-IV
Luomaranta2014 (56)	Pelvic and paraaortic nodal dissection	117	4	6	11	All type	I-IV
Nomura2006 (57)	Pelvic and paraaortic nodal dissection	155	26	4	24	All type	I-IV
Kang2014 (58)	Pelvic and paraaortic nodal dissection	397	–	–	–	All type	I-IV

### 3.2 Metastatic lymph node patterns

**Figure 2:**
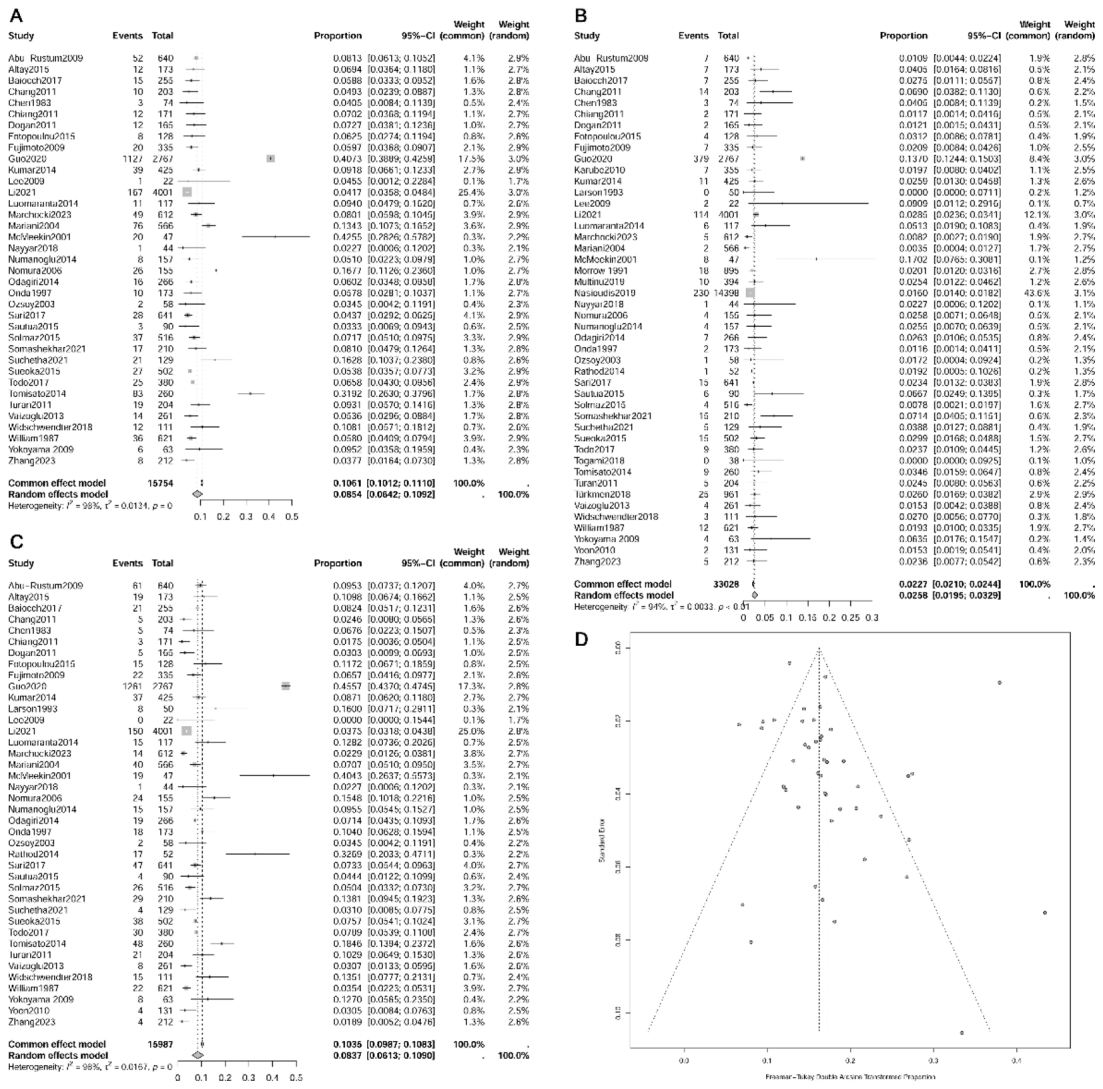
Forest plot depicting paraaortic/pelvic lymph nodes metastasis rates and funnel plot assessing publication bias. **(A)** pelvic+/para-aortic-; **(B)** pelvic-/para-aortic+; **(C)** pelvic+/para-aortic+; **(D)** funnel plot.

The results of meta-analysis revealed rates of PLN+PAN-, PLN-PAN+, and PLN+PAN+ in endometrial cancer as 8.54% (95% CI 0.0642-0.1092, I²=98%), 2.58% (95% CI 0.0195-0.0329, I²=94%), and 8.37% (95% CI 0.0613-0.1090, I²=98%), respectively (refer to **Figure 2**) (12–57). For clinical stage I endometrial cancer, meta-analysis results indicated occurrence rates of 5.92% for PLN+PAN- (95% CI 0.0258-0.1032, I²=69%), 1.00% for PLN-PAN+ (95% CI 0.0081-0.0120, I²=44%), and 2.99% for PLN+PAN+ (95% CI 0.0188-0.0431, I²=0%).

Publication bias was assessed based on the rate of PLN-PAN+, a parameter reported in most articles included. With a total of 46 studies, both the Funnel plot (see **Figure 2**) and the Egger’s test (p = 0.4401) indicated no sources of publication bias (12–57).

### 3.3 Survival outcomes of paraaortic lymph nodes metastasis

**Figure 3:**
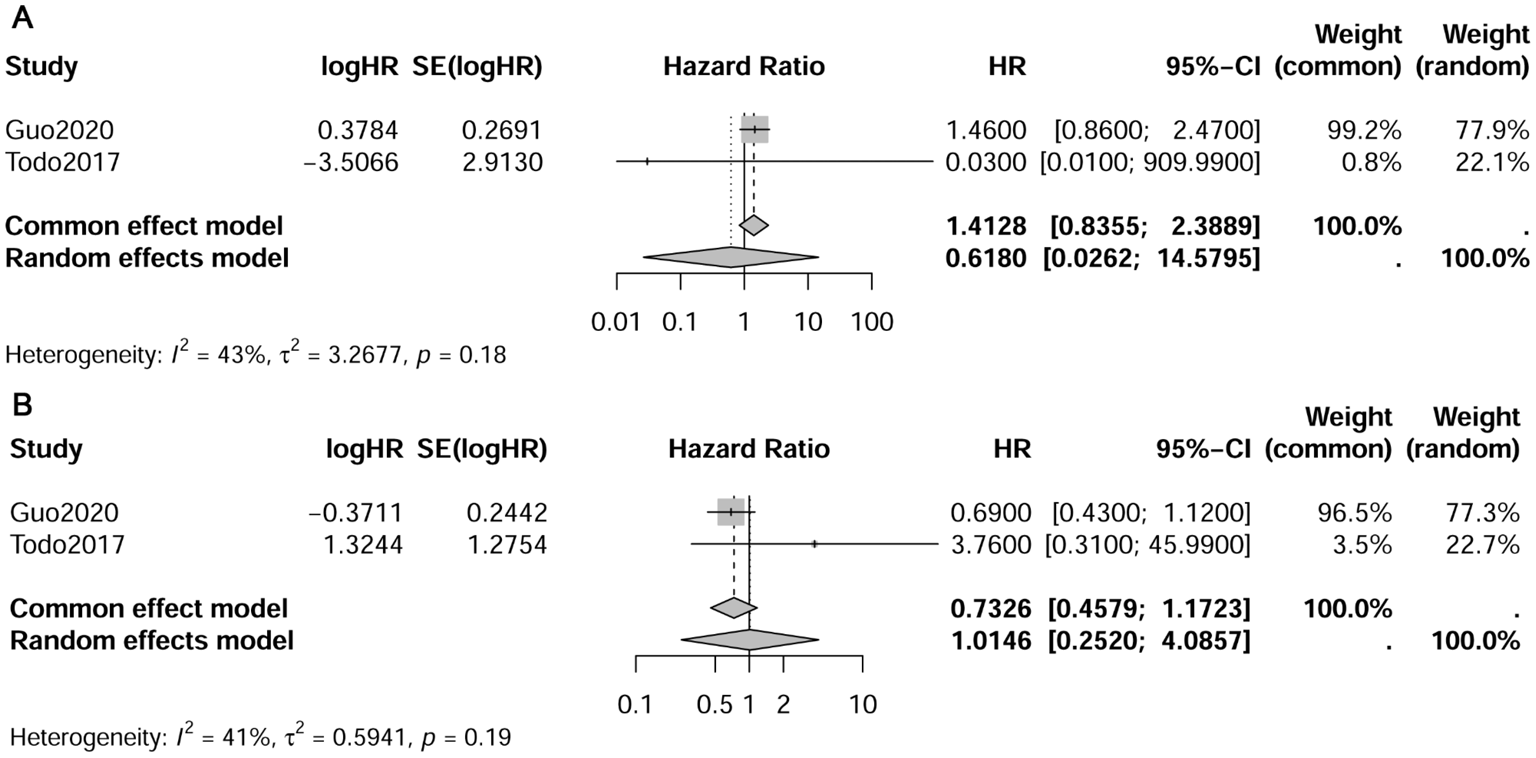
Forest plot depicting pooled survival outcomes of paraaortic lymph nodes metastasis. **(A)** pelvic-/para-aortic+ vs. pelvic+/para-aortic-; **(B)** pelvic-/para-aortic+ vs. pelvic+/para-aortic+.

In the study conducted by Todo et al., it was noted that the 5-year overall survival rates differed across various patient groups: 96.5% for PLN-PAN- patients, 77.6% for PLN+PAN - patients, 63.4% for PLN+PAN+ patients, and 53.6% for PLN-PAN+ patients (29). Our meta-analysis, however, did not reveal a significant variance in OS between PLN-PAN+ and PLN+PAN- groups (HR 1.41, 95% CI 0.84-2.39, I² = 43%). Similarly, no notable disparity in survival was observed between PLN−PAN+ and PLN+PAN+ groups (HR 0.43, 95% CI 0.46-1.12, I² = 41%) (refer to **Figure 3**) (17, 29). It’s important to highlight that this finding was derived from a meta-analysis comprising only two studies. Although survival outcomes were documented in other studies included, their data types precluded their amalgamation for meta-analysis.

The research conclusions not included in the meta-analysis generally align, indicating a consistent trend. Specifically, the survival outcomes show a decreasing trend from the PLN-PAN+ and PLN+PAN- groups to the PLN+PAN+ group. Additionally, the survival outcomes of patients with isolated paraaortic lymph node metastasis appear to be comparable to, or not inferior to, those of the PLN+PAN- group. Li et al. observed that, compared to the group PLN-PAN- group, PLN+PAN+ had the worst relapse-free survival (RFS) (HR 8.637, 95% CI 5.012-14.848, p<0.001) and disease-specific survival (DSS) (HR 15.916, 95% CI 7.817-32.404, p<0.001), followed by PLN+PAN- (RFS: HR 4.850, 95% CI 2.683-8.767, p<0.001; DSS: HR 10.635, 95% CI 5.108-22.145, p<0.001), and then PLN-PAN+ (RFS: HR 3.450, 95% CI 1.498-17.948, p=0.004; DSS: HR 6.843, 95% CI 2.378-19.697, p<0.001) (35). In the study by Tomisato et al., the 5-year progression-free survival (PFS) was reported as 87.1% for PLN−PAN− cases, 67.5% for PLN+PAN− cases, 44.4% for PLN−PAN+ cases, and 33.2% for PLN+PAN+ cases. There was no significant difference between PLN+PAN− and PLN−PAN+ (p=0.16) (32). Guo et al. identified PLN+PAN+ as an independent predictive factor for poor prognosis in patients with lymph node metastasis (HR 1.625, 95% CI 1.379-1.915, p<0.001). In patients with endometrioid tumors, PLN+PAN- and PLN-PAN+ exhibited a similar better prognosis than PLN+PAN+ in stage IIIC disease. However, in stage IIIC disease with non-endometrioid tumor and stage IV disease, there was no significant difference in survival among PLN+PAN+, PLN-PAN+ and PLN+PAN- (17).

### 3.4 Risk factors for paraaortic lymph nodes metastasis

**Figure 4:**
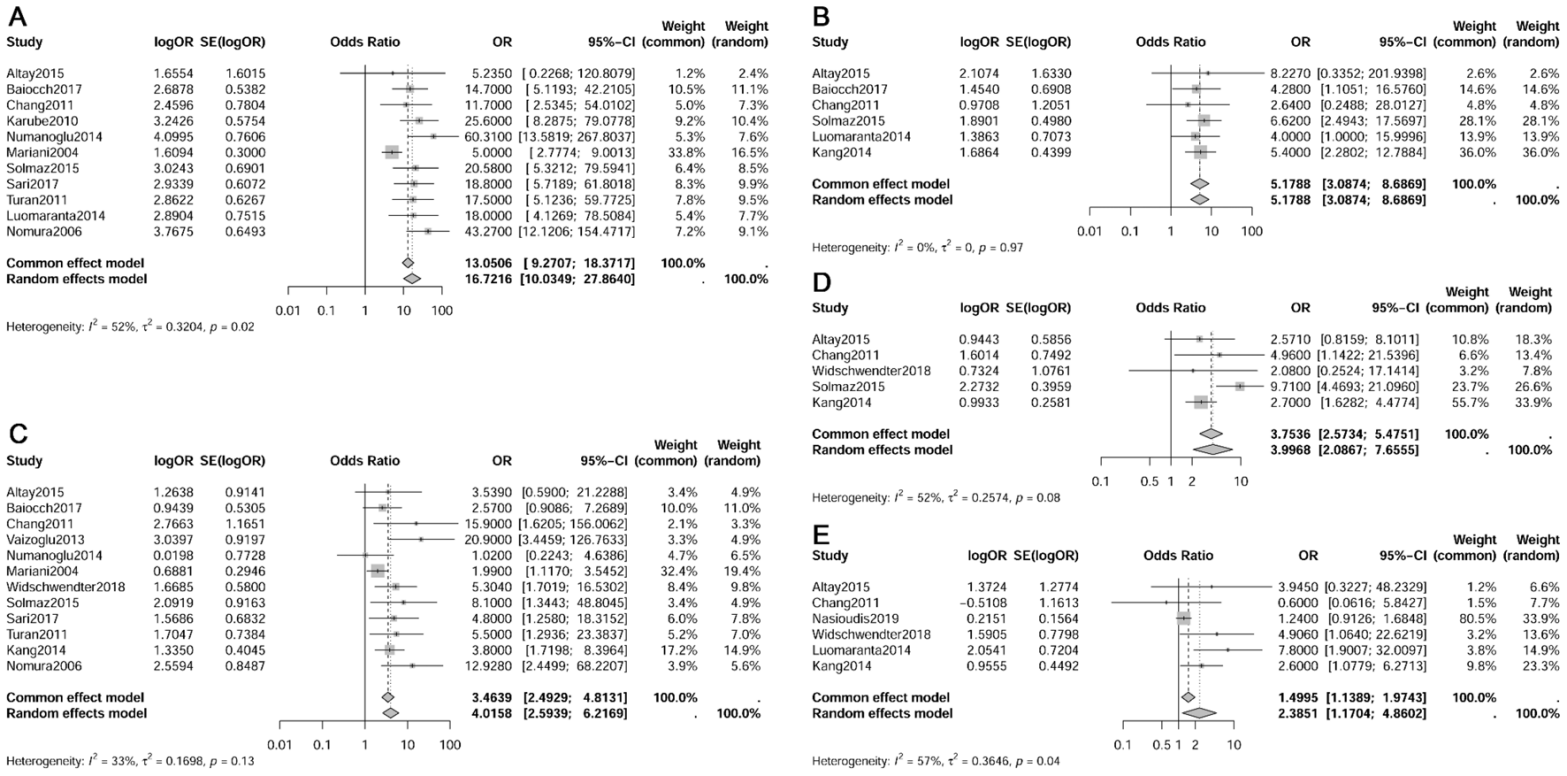
Forest plot depicting risk factors for paraaortic lymph nodes metastasis. **(A)** Pelvic lymph nodes metastasis; **(B)** Myometrial invasion; **(C)** Lymph-vascular space invasion; **(D)** Cervical invasion; **(E)** Histologic type.

The meta-analysis revealed that pelvic lymph node metastasis (OR 16.72, 95% CI 10.03-27.86, I²=52%), myometrial invasion (≥50% vs. <50%, OR 5.18, 95% CI 3.09-8.69, I²=0%), lymph-vascular space invasion (LVSI) (OR 3.46, 95% CI 2.49-4.81, I²=33%), cervical invasion (OR 4.00, 95% CI 2.09-7.66, I²=52%) and histologic type (non-endometrioid vs. endometrioid, OR 2.39, 95% CI 1.17-4.86, I²=57%) were identified as risk factors for paraaortic lymph node metastasis (refer to **Figure 4**) (13–15, 34, 36, 41, 44, 49, 52, 53, 55, 57, 58). However, histologic grade (G2 vs. G1, OR 0.81, 95% CI 0.12-5.57, I²=91%; G3 vs. G1+G2, OR 1.08, 95% CI 0.48-2.41, I²=0%), adnexal metastasis (OR 2.49, 95% CI 0.58-10.72, I²=71%), tumor size (≥2cm vs. <2cm, OR 2.86, 95% CI 0.75-10.89, I²=54%), and age (≥60 vs. <60 years, OR 0.5374, 95% CI 0.2460-1.1739, I²=0%) showed no significant association with paraaortic lymph node metastasis (13–15, 21, 34, 36, 41, 44, 49, 52, 58).

Furthermore, Change et al. reported that parametrial invasion (HR 6.15, 95% CI 0.12-329.48, p=0.371), serosal infiltration (HR 5.70, 95% CI 0.15-21068, p=0.345), positive peritoneal cytology (HR 1.24, 95% CI 0.03-51.02, p=0.910), and peritoneal seeding (HR 1.80, 95% CI 0.21-15.81, p=0.596) were not identified as risk factors for paraaortic lymph node metastasis (15). Vaizoglu et al. obtained similar results and concluded that there was no significant correlation between peritoneal cytology and serosal involvement as risk factors for paraaortic lymph node metastasis (34). Yoon et al. discovered that CA 125 levels ≥ 31 U/ml (HR 14.6, 95% CI 1.5-139.2) were independent preoperative risk factors for paraaortic lymph node metastasis (37).

## 4 Discussion

This study found that the rate of isolated paraaortic lymph node metastasis (PLN-PAN+) was found to be 2.58% (95% CI 0.0195-0.0329). The rates for PLN+PAN- and PLN+PAN+ were notably higher at 8.54% (95% CI 0.0642-0.1092) and 8.37% (95% CI 0.0613-0.1090), respectively. For clinical stage I EC, the occurrence rate was 5.92% for PLN+PAN- (95% CI 0.0258-0.1032), 1.00% for PLN-PAN+ (95% CI 0.0081-0.0120), and 2.99% for PLN+PAN+ (95% CI 0.0188-0.0431). The survival outcomes showed a gradual decline trend from PLN-PAN+ and PLN+PAN- to PLN+PAN+. Patients with isolated paraaortic lymph node metastasis exhibited comparable or non-inferior survival outcomes compared to the PLN+PAN group. The analysis indicated that pelvic lymph node metastasis (OR 16.72, 95% CI 10.03-27.86), myometrial invasion ≥50% (OR 5.18, 95% CI 3.09-8.69), lymph-vascular space invasion (LVSI) (OR 3.46, 95% CI 2.49-4.81), cervical invasion (OR 4.00, 95% CI 2.09-7.66), and non-endometrioid cancer (OR 2.39, 95% CI 1.17-4.86) were risk factors for paraaortic lymph node metastasis.

Paraaortic lymph node metastases can occur through both direct spread from pelvic metastasis and skip metastasis without involvement of pelvic lymph nodes (15). This study revealed that compared to cases of PLN+PAN- and PLN+PAN+, isolated paraaortic lymph node metastasis (PLN-PAN+) is relatively rare, accounting for 1.00% of clinical stage I endometrial cancer cases and 2.58% of all endometrial cancer cases. Multinu et al. reported a reduced rate of isolated paraaortic lymph node metastasis with the implementation of ultrastaging on negative pelvic lymph nodes during sentinel lymph node evaluation (20). The adoption of ultrastaging in sentinel lymph nodes is anticipated to improve the detection rate of low-volume pelvic metastases, including micrometastases of pelvic lymph nodes, as suggested by Guo et al. (17). Therefore, if sentinel lymph node detection is conducted in cases where routine postoperative pathological examination identifies isolated paraaortic lymph node metastasis, there is a likelihood that pelvic lymph nodes may test positive, potentially indicating pelvic involvement rather than true isolated paraaortic lymph node metastasis. It would also be interesting to know the isolated aortic involvement according to preoperative risk stratification or postoperative risk stratification in order to direct adjuvant treatments. Currently, there is a lack of relevant data on this topic, and we hope that future research will provide clearer insights into these issues.

While this meta-analysis found no significant differences in OS between the PLN-PAN+ group and the PLN+PAN- group, as well as between the PLN-PAN+ and PLN+PAN+ groups, it’s important to approach this result with caution due to the inclusion of only two meta-analysis articles. The literature generally indicates a gradual decrease in survival rates from PLN-PAN+ to PLN+PAN- and further to PLN+PAN+, even in cases where statistical differences are not observed (17, 29, 32, 35). Guo et al. identified PLN+PAN+ as an independent predictive factor for poor prognosis in patients with lymph node metastasis (17). In contrast to the notably poor survival outcomes associated with PLN+PAN+, the comparison between PLN+PAN- and PLN-PAN+ is subject to more controversy. Based on existing evidence, the PLN-PAN+ group demonstrates survival outcomes that are either similar to or not inferior to those of the PLN+PAN- group (17, 29, 32, 35). Guo et al. also found that in patients with endometrioid tumors, PLN+PAN- and PLN-PAN+ showed a better prognosis than PLN+PAN+ in stage IIIC disease (17). However, there was no significant difference in the survival rates among PLN+PAN+, PLN-PAN+, and PLN+PAN- in stage IIIC and IV diseases with non-endometrioid tumors (17). This indicates that tumor stage, pathology, and other factors can also influence the prognosis of different lymph node metastasis patterns.

Lymphadenectomy plays a pivotal role in the staging and subsequent adjuvant treatment of endometrial cancer. However, there exists controversy regarding the optimal extent of lymph node removal, particularly concerning paraaortic lymph node dissection. This debate largely revolves around weighing the benefits against the risks. A meta-analysis that included 14 studies by Pavone et al. found that pelvic and para-aortic lymphadenectomy appears to offer a prognostic advantage for women with intermediate- and high-risk endometrial cancer (59). Approximately 10% of cases involve paraaortic lymph node involvement, often coexisting with pelvic lymph node involvement (14, 49). This meta-analysis has identified pelvic lymph node metastasis as a significant risk factor for paraaortic lymph node metastasis. Studies have reported that 28.6–66.7% of patients with pelvic lymph node metastasis also exhibit paraaortic node involvement (51, 52). Luomaranta et al. discovered that the presence of grossly positive pelvic nodes correlated with the prediction of para-aortic metastasis, demonstrating a sensitivity of 52.4% and a specificity of 93.8% (56). Despite increased intraoperative complications associated with paraaortic lymph node dissection, survival benefits have been noted in cases where positive pelvic lymph node metastasis exists. A retrospective study revealed that among patients with positive pelvic lymph nodes undergoing postoperative radiation therapy, those who underwent concurrent paraaortic lymph node dissection experienced improved survival rates and paraaortic recurrence control compared to those who did not undergo the procedure (37). The recommended extent of paraaortic lymphadenectomy extends to the level of the renal vessels due to the substantial presence of metastatic lymph nodes above the inferior mesenteric artery (3). Nevertheless, isolated paraaortic lymph node metastasis, while relatively infrequent (2.58% as indicated in this meta-analysis), underscores the need for cautious consideration even in patients with negative pelvic lymph nodes. Identifying additional risk factors for positive paraaortic lymph nodes and conducting targeted dissections represent the most optimal approach to addressing this issue.

In addition to pelvic lymph node metastasis, this meta-analysis identified that LVSI, myometrial invasion ≥50%, cervical invasion, and non-endometrioid EC are also significant risk factors for paraaortic lymph node metastasis. Sari et al. recommended LVSI as a crucial predictor in intraoperative frozen section analysis to guide paraaortic lymphadenectomy (53). Chang et al. reported LVSI as the sole independent factor for isolated paraaortic lymph node metastasis (15). The incidence rate of paraaortic lymph node metastasis was only 0.5%-0.8% among patients without LVSI (34, 53). Solmaz et al. suggested combining pelvic lymph node status with LVSI status to predict paraaortic lymph node involvement; when both pathological markers were negative, the incidence of paraaortic lymph node metastasis was only 0.1% (52).

When multiple risk factors converge, surgical decisions may become more prone to controversy. ESGO guidelines recommend both pelvic and para-aortic lymphadenectomy for stage II endometrial cancer (60). Kumar et al. recommended paraaortic lymphadenectomy in type II histology endometrial cancer due to a 5% occurrence of isolated paraaortic metastasis even when myometrial infiltration is ≤50% (43). On the other hand, Baiocchi et al. reported that paraaortic lymphadenectomy can be omitted in patients with superficial myometrial invasion and negative pelvic lymph node metastasis, with less than 1% risk of paraaortic lymph node metastasis, irrespective of histological type (14).

Another challenge lies in determining targeted dissection based on predictive factors of positive paraaortic lymph nodes. It is optimal to determine predictive factors before or during surgery. While factors such as lymph node metastasis, LVSI, myometrial invasion, and histologic type can be suggested preoperatively and intraoperatively, ensuring their accuracy in alignment with the final postoperative pathological results remains a concern (53).

Despite efforts to identify predictors preoperatively and intraoperatively, accurately determining clinical and pathological factors before final pathology remains challenging, and errors in frozen section analysis may occur. Further studies are needed to develop more accurate predictors and improve patient selection for paraaortic lymphadenectomy. Despite following a rigorous review protocol for study selection, data extraction, and analysis, this study has several limitations. Firstly, the retrospective nature of included studies inherently carries limitations. Secondly, subgroup analyses lacked uniform standards, precluding meta-analysis. Thirdly, for survival meta-analysis, only a limited number of studies were included. Additionally, the use of a random-effects model in some meta-analyses may introduce variability in weighting large studies during statistical heterogeneity, potentially impacting the combined results. These limitations warrant cautious interpretation of the study’s findings.

In summary, isolated paraaortic lymph node metastasis, though relatively rare, can still occur even in clinical stage I endometrial cancer. The survival outcomes of patients with isolated paraaortic lymph node metastasis appear to be comparable to, or not inferior to, those of the PLN+PAN- group. Even in patients with negative pelvic lymph nodes, careful consideration should be given to the possibility of para-aortic lymph node metastasis, especially in those with high-risk factors.

## References

[1] Siegel RL Miller KD Wagle NS Jemal A . Cancer statistics, 2023. CA Cancer J Clin. (2023) 73:17–48. doi: 10.3322/caac.21763 36633525

[2] Abu-Rustum N Yashar C Arend R Barber E Bradley K Brooks R . Uterine neoplasms, version 1.2023, NCCN clinical practice guidelines in oncology. J Natl Compr Canc Netw. (2023) 21:181–209. doi: 10.6004/jnccn.2023.0006 36791750

[3] Crosbie EJ Kitson SJ McAlpine JN Mukhopadhyay A Powell ME Singh N . Endometrial cancer. Lancet. (2022) 399:1412–28. doi: 10.1016/S0140-6736(22)00323-3 35397864

[4] FIGO committee on gynecologic oncology . Revised FIGO staging for carcinoma of the vulva, cervix, and endometrium. Int J Gynaecol Obstet. (2009) 105:103–4. doi: 10.1016/j.ijgo.2009.02.012 19367689

[5] Li L Tang M Nie D Gou J Li Z . Para-aortic lymphadenectomy did not improve overall survival among women with type I endometrial cancer. Int J Gynaecol Obstet. (2020) 150:163–8. doi: 10.1002/ijgo.13228 32433783

[6] Lai YL Chang CS Chang K Kim HS Chen J Cheng WF . Does para-aortic lymphadenectomy improve survival in pathologically diagnosed early-stage grade 3 endometrioid and non-endometrioid endometrial cancers? A retrospective cohort study in Korea and Taiwan. Gynecol Oncol. (2022) 167:65–72. doi: 10.1016/j.ygyno.2022.08.009 35995599

[7] Todo Y Kato H Kaneuchi M Watari H Takeda M Sakuragi N . Survival effect of para-aortic lymphadenectomy in endometrial cancer (SEPAL study): a retrospective cohort analysis. Lancet. (2010) 375:1165–72. doi: 10.1016/S0140-6736(09)62002-X 20188410

[8] Restaino S Poli A Arcieri M Mariuzzi L Orsaria M Tulisso A . Molecular classification of endometrial carcinoma on endometrial biopsy: an early prognostic value to guide personalized treatment. Int J Gynecol Cancer. (2024) 34:1211–6. doi: 10.1136/ijgc-2024-005478 38955372

[9] Bodurtha Smith AJ Fader AN Tanner EJ . Sentinel lymph node assessment in endometrial cancer: a systematic review and meta-analysis. Am J Obstet Gynecol. (2017) 216:459–476.e10. doi: 10.1016/j.ajog.2016.11.1033 27871836

[10] Holloway RW Abu-Rustum NR Backes FJ Boggess JF Gotlieb WH Jeffrey Lowery W . Sentinel lymph node mapping and staging in endometrial cancer: A Society of Gynecologic Oncology literature review with consensus recommendations. Gynecol Oncol. (2017) 146:405–15. doi: 10.1016/j.ygyno.2017.05.027 PMC6075736 28566221

[11] Liberati A Altman DG Tetzlaff J Mulrow C Gøtzsche PC Ioannidis JP . The PRISMA statement for reporting systematic reviews and meta-analyses of studies that evaluate healthcare interventions: explanation and elaboration. J Clin Epidemiol. (2009) 62:e1–34. doi: 10.1016/j.jclinepi.2009.06.006 19631507

[12] Abu-Rustum NR Gomez JD Alektiar KM Soslow RA Hensley ML Leitao MM Jr . The incidence of isolated paraaortic nodal metastasis in surgically staged endometrial cancer patients with negative pelvic lymph nodes. Gynecol Oncol. (2009) 115:236–8. doi: 10.1016/j.ygyno.2009.07.016 19666190

[13] Altay A Toptas T Dogan S Simsek T Pestereli E . Analysis of metastatic regional lymph node locations and predictors of para-aortic lymph node involvement in endometrial cancer patients at risk for lymphatic dissemination. Int J Gynecol Cancer. (2015) 25:657–64. doi: 10.1097/IGC.0000000000000392 25647255

[14] Baiocchi G Faloppa CC Mantoan H Camarço WR Badiglian-Filho L Kumagai LY . Para-aortic lymphadenectomy can be omitted in most endometrial cancer patients at risk of lymph node metastasis. J Surg Oncol. (2017) 116:220–6. doi: 10.1002/jso.24651 28482122

[15] Chang SJ Kong TW Kim WY Yoo SC Yoon JH Chang KH . Lymph-vascular space invasion as a significant risk factor for isolated para-aortic lymph node metastasis in endometrial cancer: a study of 203 consecutive patients. Ann Surg Oncol. (2011) 18:58–64. doi: 10.1245/s10434-010-1206-x 20607418

[16] Chiang AJ Yu KJ Chao KC Teng NN . The incidence of isolated para-aortic nodal metastasis in completely staged endometrial cancer patients. Gynecol Oncol. (2011) 121:122–5. doi: 10.1016/j.ygyno.2010.11.026 21194737

[17] Guo J Qian H Ma F Zhang Y Cui X Duan H . The characteristics of isolated para-aortic lymph node metastases in endometrial cancer and their prognostic significance. Ther Adv Med Oncol. (2020) 12:1758835920933036. doi: 10.1177/1758835920933036 32587635 PMC7294490

[18] Lee JH Jung US Kyung MS Hoh JK Choi JS . Laparoscopic systemic retroperitoneal lymphadenectomy for women with low-risk early endometrial cancer. Ann Acad Med Singap. (2009) 38:581–6. doi: 10.47102/annals-acadmedsg.v38n7p581 19652848

[19] Marchocki Z Cusimano MC Vicus D Pulman K Rouzbahman M Mirkovic J . Diagnostic accuracy of frozen section and patterns of nodal spread in high grade endometrial cancer: A secondary outcome of the SENTOR prospective cohort study. Gynecol Oncol. (2023) 173:41–8. doi: 10.1016/j.ygyno.2023.04.004 37075495

[20] Multinu F Casarin J Cappuccio S Keeney GL Glaser GE Cliby WA . Ultrastaging of negative pelvic lymph nodes to decrease the true prevalence of isolated paraaortic dissemination in endometrial cancer. Gynecol Oncol. (2019) 154:60–4. doi: 10.1016/j.ygyno.2019.05.008 PMC6612056 31126637

[21] Nasioudis D Holcomb K . Incidence of isolated para-aortic lymph node metastasis in early stage endometrial cancer. Eur J Obstet Gynecol Reprod Biol. (2019) 242:43–6. doi: 10.1016/j.ejogrb.2019.09.003 31557556

[22] Nayyar N Lakhwani P Goel A Pande PK Kumar K . The futility of systematic lymphadenectomy in early-stage low-grade endometrial cancer. Indian J Surg Oncol. (2018) 9:204–10. doi: 10.1007/s13193-018-0753-7 PMC5984865 29887702

[23] Odagiri T Watari H Kato T Mitamura T Hosaka M Sudo S . Distribution of lymph node metastasis sites in endometrial cancer undergoing systematic pelvic and para-aortic lymphadenectomy: a proposal of optimal lymphadenectomy for future clinical trials. Ann Surg Oncol. (2014) 21:2755–61. doi: 10.1245/s10434-014-3663-0 24705578

[24] Ozsoy M Dilek S Ozsoy D . Pelvic and paraaortic lymph node metastasis in clinical stage I endometrial adenocarcinoma: an analysis of 58 consecutive cases. Eur J Gynaecol Oncol. (2003) 24:398–400. 14584655

[25] Rathod PS Shakuntala PN Pallavi VR Kundaragi R Shankaranand B Vijay CR . The risk and pattern of pelvic and para aortic lymph nodal metastasis in patients with intermediate and high risk endometrial cancer. Indian J Surg Oncol. (2014) 5:109–14. doi: 10.1007/s13193-014-0303-x PMC4116541 25114462

[26] Sautua RR Goiri K Calle MA Marin IJ Artola AL . Incidence of nodal metastasis and isolated aortic metastases in patients with surgically staged endometrioid endometrial cancer. Int J Gynecol Cancer. (2015) 25:875–8. doi: 10.1097/IGC.0000000000000428 25774712

[27] Somashekhar SP Rohit Kumar C Anil J Vijay A Rakshit SH Ashwin KR . Prospective non-randomized control trial on role of systematic high para-aortic lymphadenectomy in endometrial cancer: Indian study. Indian J Gynecol Oncol. (2021) 19:6. doi: 10.1007/s40944-020-00482-9

[28] Suchetha S Mathew AP Rema P Thomas S . Pattern of lymph node metastasis in endometrial cancer: a single institution experience. Indian J Surg Oncol. (2021) 12:73–7. doi: 10.1007/s13193-020-01227-y PMC7960813 33814835

[29] Todo Y Takeshita S Okamoto K Yamashiro K Kato H . Implications of para-aortic lymph node metastasis in patients with endometrial cancer without pelvic lymph node metastasis. J Gynecol Oncol. (2017) 28:e59. doi: 10.3802/jgo.2017.28.e59 28657221 PMC5540719

[30] Sueoka K Umayahara K Abe A Usami T Yamamoto A Nomura H . Prognosis for endometrial cancer patients treated with systematic pelvic and para-aortic lymphadenectomy followed by platinum-based chemotherapy. Int J Gynecol Cancer. (2015) 25:81–6. doi: 10.1097/IGC.0000000000000268 25347094

[31] Togami S Kawamura T Fukuda M Yanazume S Kamio M Kobayashi H . Prospective study of sentinel lymph node mapping for endometrial cancer. Int J Gynaecol Obstet. (2018) 143:313–8. doi: 10.1002/ijgo.12651 30125949

[32] Tomisato S Yamagami W Susumu N Kuwahata M Takigawa A Nomura H . Clinicopathological study on para-aortic lymph node metastasis without pelvic lymph node metastasis in endometrial cancer. J Obstet Gynaecol Res. (2014) 40:1733–9. doi: 10.1111/jog.12399 24888941

[33] Türkmen O Başaran D Karalök A Cömert Kimyon G Taşçı T Üreyen I . Prognostic effect of isolated paraaortic nodal spread in endometrial cancer. J Turk Ger Gynecol Assoc. (2018) 19:201–5. doi: 10.4274/jtgga.2017.0152 PMC6250084 29588264

[34] Vaizoglu F Yuce K Salman MC Basaran D Calis P Ozgul N . Lymphovascular space involvement is the sole independent predictor of lymph node metastasis in clinical early stage endometrial cancer. Arch Gynecol Obstet. (2013) 288:1391–7. doi: 10.1007/s00404-013-2913-x 23764931

[35] Li W Jiang J Fu Y Shen Y Zhang C Yao S . Implications of isolated para-aortic lymph node metastasis in endometrial cancer: A large-scale, multicenter, and retrospective study. Front Med (Lausanne). (2021) 8:754890. doi: 10.3389/fmed.2021.754890 34746191 PMC8566710

[36] Widschwendter P Bauer E De Gregorio N Bekes I Janni W Scholz C . Influence of prognostic factors on lymph node involvement in endometrial cancer: A single-center experience. Int J Gynecol Cancer. (2018) 28:1145–52. doi: 10.1097/IGC.0000000000001290 29757871

[37] Yoon JH Yoo SC Kim WY Chang SJ Chang KH Ryu HS . Para-aortic lymphadenectomy in the management of preoperative grade 1 endometrial cancer confined to the uterine corpus. Ann Surg Oncol. (2010) 17:3234–40. doi: 10.1245/s10434-010-1199-5 20585865

[38] Zhang X Chen S Li G Zheng L Shang S Li J . Investigating the influence of primary uterine tumor site on pelvic and para-aortic lymph node metastatic pattern and evaluating the risk factors for lymph node metastases in endometrial carcinoma: A retrospective study. Med (Baltimore). (2023) 102:e36100. doi: 10.1097/MD.0000000000036100 PMC10681575 38013262

[39] Creasman WT Morrow CP Bundy BN Homesley HD Graham JE Heller PB . Surgical pathologic spread patterns of endometrial cancer. A Gynecologic Oncol Group Study Cancer. (1987) 60:2035–41. doi: 10.1002/1097-0142(19901015)60:8+<2035::aid-cncr2820601515>3.0.co;2-8 3652025

[40] Dogan NU Gungor T Karsli F Ozgu E Besli M . To what extent should para-aortic lymphadenectomy be carried out for surgically staged endometrial cancer? Int J Gynecol Cancer. (2012) 22:607–10. doi: 10.1097/IGC.0b013e3182434adb 22546819

[41] Karube Y Fujimoto T Takahashi O Nanjyo H Mizunuma H Yaegashi N . Histopathological prognostic factors predicting para-aortic lymph node metastasis in patients with endometrioid uterine cancer. Gynecol Oncol. (2010) 118:151–4. doi: 10.1016/j.ygyno.2010.05.004 20621776

[42] Onda T Yoshikawa H Mizutani K Mishima M Yokota H Nagano H . Treatment of node-positive endometrial cancer with complete node dissection, chemotherapy and radiation therapy. Br J Cancer. (1997) 75:1836–41. doi: 10.1038/bjc.1997.313 PMC2223619 9192991

[43] Kumar S Podratz KC Bakkum-Gamez JN Dowdy SC Weaver AL McGree ME . Prospective assessment of the prevalence of pelvic, paraaortic and high paraaortic lymph node metastasis in endometrial cancer. Gynecol Oncol. (2014) 132:38–43. doi: 10.1016/j.ygyno.2013.10.002 24120926 PMC4381544

[44] Numanoglu C Corbacioglu Esmer A Ulker V Goksedef BP Han A Akbayir O . The prediction of para-aortic lymph node metastasis in endometrioid adenocarcinoma of endometrium. J Obstet Gynaecol. (2014) 34:177–81. doi: 10.3109/01443615.2013.844112 24456443

[45] Chen SS Lee L . Retroperitoneal lymph node metastases in Stage I carcinoma of the endometrium: correlation with risk factors. Gynecol Oncol. (1983) 16:319–25. doi: 10.1016/0090-8258(83)90157-9 6654176

[46] Morrow CP Bundy BN Kurman RJ Creasman WT Heller P Homesley HD . Relationship between surgical-pathological risk factors and outcome in clinical stage I and II carcinoma of the endometrium: a Gynecologic Oncology Group study. Gynecol Oncol. (1991) 40:55–65. doi: 10.1016/0090-8258(91)90086-k 1989916

[47] Fujimoto T Nanjyo H Fukuda J Nakamura A Mizunuma H Yaegashi N . Endometrioid uterine cancer: histopathological risk factors of local and distant recurrence. Gynecol Oncol. (2009) 112:342–7. doi: 10.1016/j.ygyno.2008.10.019 19062082

[48] Larson DM Johnson KK . Pelvic and para-aortic lymphadenectomy for surgical staging of high-risk endometrioid adenocarcinoma of the endometrium. Gynecol Oncol. (1993) 51:345–8. doi: 10.1006/gyno.1993.1301 8112643

[49] Mariani A Keeney GL Aletti G Webb MJ Haddock MG Podratz KC . Endometrial carcinoma: paraaortic dissemination. Gynecol Oncol. (2004) 92:833–8. doi: 10.1016/j.ygyno.2003.11.032 14984949

[50] McMeekin DS Lashbrook D Gold M Scribner DR Kamelle S Tillmanns TD . Nodal distribution and its significance in FIGO stage IIIc endometrial cancer. Gynecol Oncol. (2001) 82:375–9. doi: 10.1006/gyno.2001.6278 11531298

[51] Yokoyama Y Maruyama H Sato S Saito Y . Indispensability of pelvic and paraaortic lymphadenectomy in endometrial cancers. Gynecol Oncol. (1997) 64:411–7. doi: 10.1006/gyno.1996.4573 9062142

[52] Solmaz U Mat E Dereli ML Turan V Tosun G Dogan A . Lymphovascular space invasion and positive pelvic lymph nodes are independent risk factors for para-aortic nodal metastasis in endometrioid endometrial cancer. Eur J Obstet Gynecol Reprod Biol. (2015) 186:63–7. doi: 10.1016/j.ejogrb.2015.01.006 25638600

[53] Sari ME Yalcin İ Sahin H Meydanli MM Gungor T . Risk factors for paraaortic lymph node metastasis in endometrial cancer. Int J Clin Oncol. (2017) 22:937–44. doi: 10.1007/s10147-017-1139-5 28523533

[54] Fotopoulou C El-Balat A du Bois A Sehouli J Harter P Muallem MZ . Systematic pelvic and paraaortic lymphadenectomy in early high-risk or advanced endometrial cancer. Arch Gynecol Obstet. (2015) 292:1321–7. doi: 10.1007/s00404-015-3746-6 25990476

[55] Turan T Hizli D Sarici S Boran N Gundogdu B Karadag B . Is it possible to predict para-aortic lymph node metastasis in endometrial cancer? Eur J Obstet Gynecol Reprod Biol. (2011) 158:274–9. doi: 10.1016/j.ejogrb.2011.04.031 21664758

[56] Luomaranta A Lohi J Bützow R Leminen A Loukovaara M . Prediction of para-aortic spread by gross pelvic lymph node findings in patients with endometrial carcinoma. Int J Gynecol Cancer. (2014) 24:697–702. doi: 10.1097/IGC.0000000000000113 24662132

[57] Nomura H Aoki D Suzuki N Susumu N Suzuki A Tamada Y . Analysis of clinicopathologic factors predicting para-aortic lymph node metastasis in endometrial cancer. Int J Gynecol Cancer. (2006) 16:799–804. doi: 10.1111/j.1525-1438.2006.00529.x 16681764

[58] Kang S Lee JM Lee JK Kim JW Cho CH Kim SM . A Web-based nomogram predicting para-aortic nodal metastasis in incompletely staged patients with endometrial cancer: a Korean Multicenter Study. Int J Gynecol Cancer. (2014) 24:513–9. doi: 10.1097/IGC.0000000000000090 24552891

[59] Pavone M Jochum F Lecointre L Fanfani F Scambia G Querleu D . Therapeutic role of para-aortic lymphadenectomy in patients with intermediate- and high-risk endometrial cancer: a systematic review and meta-analysis. Int J Gynecol Cancer. (2024) 34:519–27. doi: 10.1136/ijgc-2023-005134 38296516

[60] Concin N Matias-Guiu X Vergote I Cibula D Mirza MR Marnitz S . ESGO/ESTRO/ESP guidelines for the management of patients with endometrial carcinoma. Int J Gynecol Cancer. (2021) 31:12–39. doi: 10.1136/ijgc-2020-002230 33397713

